# O-GlcNAcylation regulates extracellular signal-regulated kinase (ERK) activation in Alzheimer’s disease

**DOI:** 10.3389/fnagi.2023.1155630

**Published:** 2023-07-04

**Authors:** Sophiya John Ephrame, Gentry K. Cork, Victoria Marshall, Margaret A. Johnston, Jenna Shawa, Ibtihal Alghusen, Amy Qiang, Aspin R. Denson, Marisa S. Carman, Halyna Fedosyuk, Russell H. Swerdlow, Chad Slawson

**Affiliations:** ^1^Department of Biochemistry and Molecular Biology, University of Kansas Medical Center, Kansas City, KS, United States; ^2^School of Medicine, University of Kansas Medical Center, Kansas City, KS, United States; ^3^Department of Neurology, University of Kansas Medical Center, Kansas City, KS, United States; ^4^University of Kansas Alzheimer’s Disease Research Center, University of Kansas Medical Center, Kansas City, KS, United States

**Keywords:** O-GlcNAc, OGT, OGA, ERK, APP, Alzheimer’s disease, Thiamet-G

## Abstract

**Introduction:**

Aberrant activation of Extracellular Signal-Regulated Kinase (ERK) signaling is associated with Alzheimer’s disease (AD) pathogenesis. For example, enhanced ERK signal activation mediated by Apolipoprotein E4 (APOE4), which is a critical genetic risk factor for AD, increases the transcription of amyloid precursor protein (APP). We hypothesize that O-linked N-acetylglucosamine (O-GlcNAc) regulates the phosphorylation and activation of ERK. O-GlcNAc is a single sugar post-translational modification that dynamically cycles on and off proteins in response to nutrient changes by the action of the enzymes O-GlcNAc transferase (OGT) and O-GlcNAcase (OGA), respectively. However, O-GlcNAc quickly returns to a baseline level after stimulus removal (called O-GlcNAc homeostasis).

**Methods:**

We did a serum reactivation time-course followed by western blot in SH-SY5Y neuroblastoma cells after long-term O-GlcNAcase (OGA) inhibition by Thiamet-G (TMG) treatment, O-GlcNAc transferase (OGT) knock-down (KD) and OGA KD. Brain tissues of C57BL6/J mice and 5XFAD Alzheimer’s disease mice intra-peritoneally injected with TMG for 1 month and C57BL6/J mice intra-peritoneally injected with TMG for 6 months were also used for western blot.

**Results:**

We found that ERK1/2 phosphorylation at Thr 202/Tyr204 and Thr183/Tyr185 (p-ERK) are amplified and hence ERK1/2 are activated after long-term OGA inhibition in SH-SY5Y cells. In addition to pharmacological treatment, genetic disruption of O-GlcNAc by OGT KD and OGA KD also increased p-ERK in SH-SY5Y cells suggesting O-GlcNAc homeostasis controls ERK signaling. To determine how O-GlcNAc regulates p-ERK, we probed the expression of phosphorylated mitogen-activated protein kinase-kinase (p-MEK) which phosphorylates and activates ERK and Dual specificity phosphatase-4 (DUSP4) which dephosphorylates and inactivates ERK in SH-SY5Y cells. p-MEK increases in TMG treated and OGT KD cells whereas total DUSP4 decreases in OGT KD and OGA KD cells with serum reactivation time course. Next, we probed the role of OGA inhibition in regulating ERK activation using mice brain-tissue samples. Interestingly, 6-month intra-peritoneal TMG injection in C57BL/6J mice showed an increase in amplitude of p-ERK and APP protein levels, indicating long-term OGA inhibition potentially contributes to AD progression. Furthermore, 1-month TMG injection was sufficient to increase the amplitude of p-ERK in 5XFAD AD mice brains suggesting AD phenotype contributes to the acceleration of ERK activation mediated by OGA inhibition.

**Conclusion:**

Together, these results indicate that disruptions to O-GlcNAc homeostasis amplify ERK signal activation in AD.

## Introduction

**FIGURE 1:**
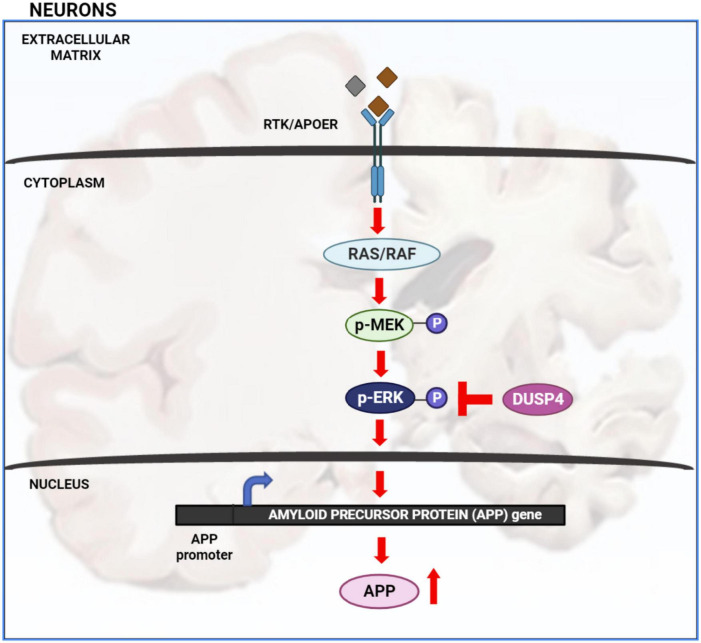
Extracellular signal-regulated kinase (ERK) signaling cascade in AD: activation of receptor tyrosine kinase (RTK) or APOE receptor (APOER) leads to the activation of Ras/Raf protein which in turn phosphorylates and activates MEK. MEK phosphorylates and activates ERK. Once ERK is activated it activates numerous downstream targets both in the cytosol and nucleus. Activation of nuclear transcription factors by ERK leads to increased transcription of APP gene and hence increased APP protein production. Increased level of APP protein potentially leads to increased Aβ plaque formation. ERK is inactivated by de-phosphorylation via dual specificity phosphatases such as DUSP4.

Extracellular signal-regulated kinase (ERK) signaling plays a significant role in neuronal development and memory formation. However, hyper-activation of ERK is associated with Alzheimer’s disease (AD) pathogenesis (Mills et al., 1997; Huang et al., 2017, 2019; Kirouac et al., 2017). For example, the transcription of amyloid precursor protein (APP) is increased through non-canonical ERK signaling activated by Apolipoprotein E4 (APOE4), a critical genetic risk factor for AD. Binding of APOE4 to the APOE receptor leads to ERK1 activation by upstream kinases such as dual leucine-zipper kinase (DLK) and MAP kinase kinase 7 (MKK7). ERK phosphorylates and activates the transcription factor c-Fos which in turn binds to the APP promoter increasing APP gene transcription associated with Aβ plaque accumulation (Huang et al., 2017). Thus, understanding how ERK signaling is regulated is crucial for understanding AD pathology (Figure 1).

Interestingly, ERK signaling was predicted to be significantly upregulated via Ingenuity Pathway Analysis (IPA) of RNA sequencing data in SH-SY5Y neuroblastoma cells after increased O-GlcNAcylation from long-term treatment with O-GlcNAcase (OGA) inhibitor Thiamet-G (TMG) (Tan et al., 2017). O-GlcNAcylation is a type of post-translational modification involving the attachment of a single N-acetylglucosamine (GlcNAc) sugar by O-GlcNAc transferase (OGT) to serine or threonine residues of nuclear, cytosolic, and mitochondrial proteins (Hart et al., 2011; Zhang et al., 2014). Multiple nutrients such as glucose, amino acids, fatty acids, and nucleotides feed into the Hexosamine Biosynthetic Pathway (HBP) to produce UDP-GlcNAc, the nutrient sensitive metabolic substrate for OGT. Furthermore, the modification is dynamically cycled off substrates by the actions of O-GlcNAcase (OGA) (Hart et al., 2011; Zhang et al., 2014; Machacek et al., 2018).

Although O-GlcNAc cycling is an essential response to environmental changes (Zachara and Hart, 2004), O-GlcNAc levels quickly return to baseline after stimulus removal, termed O-GlcNAc homeostasis. O-GlcNAc homeostasis is disrupted in several diseases including AD (Zhu et al., 2003; Liu et al., 2009; Förster et al., 2014), but the exact role of O-GlcNAc homeostasis in AD is under debate. O-GlcNAcylation, OGT, and OGA expression was measured in nine age and sex matched control and AD post-mortem human brains finding an increase in O-GlcNAc levels and a decline in OGA expression in AD (Förster et al., 2014). Griffith and Schmitz (1995) also showed O-GlcNAcylation is upregulated in AD brains. Furthermore, a quantitative proteomics study using 10 control and 10 post-mortem AD brains identified 119 O-GlcNAc peptides that showed increased abundance in AD suggesting loss of OGA expression with AD progression would increase O-GlcNAc on proteins (Wang et al., 2017). However, some groups report a decrease in total O-GlcNAc levels from post-mortem AD brains (Liu et al., 2009; Wang et al., 2016). For example, protein O-GlcNAcylation was markedly decreased in AD cerebrum from seven AD cases and seven control (Liu et al., 2009). Therefore, it is unclear if O-GlcNAc increases or decreases in AD, and the role of O-GlcNAc in AD is in dispute.

Alternatively, increasing tau O-GlcNAcylation by OGA inhibition is associated with decreased tau phosphorylation (Yuzwa et al., 2008, 2012; Liu et al., 2009). Hyper-phosphorylation of tau at proline directed serine/threonine phosphorylation sites leads to neurofibrillary tangle formation, which is another hallmark of AD along with Aβ plaque accumulation (Yuzwa et al., 2008; Selnick et al., 2019; Shcherbinin et al., 2020; Permanne et al., 2022; Yin et al., 2022). Interestingly, TMG decreased pathological tau phosphorylation at Thr231, Ser396 and Ser422 in rat cortices and hippocampi (Yuzwa et al., 2008). Therefore, at least three unique OGA inhibitors from AscenNeuron, Eli Lilly, and Merck are currently in Phase I or II clinical trials to increase O-GlcNAcylation of tau and prevent tau phosphorylation (Selnick et al., 2019; Shcherbinin et al., 2020; Permanne et al., 2022). For example, phosphorylation of tau at Ser-202/Thr-205 (AT8 antibody site), and Ser-214 and/or Ser-212 (AT100 antibody site) decreased in the dentate gyrus (granular cell layer) of transgenic P301S tau mice upon chronic treatment (3.5 months) with the OGA inhibitor ASN90 from AscenNeuron (Permanne et al., 2022).

In our current study, we show that disruptions to O-GlcNAc homeostasis by OGA inhibition, OGA KD, and OGT knock-down (KD) all increase ERK phosphorylation in SH-SY5Y neuroblastoma cells. Additionally, 6-month intraperitoneal TMG injection increased ERK phosphorylation along with APP in C57BL6J mice brains, and 1-month TMG injection increased ERK phosphorylation in 5XFAD (Familial Alzheimer’s Disease) mice. These data suggest that alterations to O-GlcNAc homeostasis increase the amplitude of ERK activation associated with AD pathogenesis.

## Materials and methods

### Cell culture

SH-SY5Y neuroblastoma cells and HeLa cervical cancer cells were cultured in DMEM (Sigma D5030-10L) with 4 mM Glucose supplemented with 44 mM sodium bicarbonate (Sigma), 15 mg/liter phenol red (Sigma) and with 10% fetal bovine serum (FBS; Gemini), 1% penicillin/streptomycin (Sigma), and 1% GlutaMAX (Gibco). DMEM media was changed every day. The cells were treated with 10 μM Thiamet-G (SD Specialty Chemicals) (TMG, from 20 mM stock, Tris-Buffered Saline pH 7.4) for at least 2 weeks prior to experiments. These cells were serum starved overnight and re-stimulated the next day to induce a robust activation of ERK signaling. Cells were harvested at 0, 5, 10, 20, 60, and 120 min upon serum re-activation (Tan et al., 2017; Machacek et al., 2018). The cells were harvested without serum reactivation to measure APP protein levels.

### Lentivirus preparation

The OGT knock-down (KD) and OGA KD cells were generated by sh-RNA mediated lentiviral KD (ThermoFisher). Plasmids of OGT KD shRNAs, OGA KD shRNAs, and scramble GFP shRNA, along with plasmids that encode for lentiviral particles, were purified using maxiprep kit (NA0310 – Sigma Aldrich). HEK293T cells were seeded at a density of 5×(10^6) cells in each 10 cm dish having a total volume of 10 ml DMEM (25mM Glucose) during day 1. During day 2, each HEK293T cell-plate was transfected using TransIT-X2^®^ Dynamic Delivery System (Mirus MIR 6005) in 1.5 mL of opti-MEM serum-free media (ThermoFisher Catalog # 11058021) by adding OGT or OGA shRNA plasmids along with PCMV and PMD2G plasmids encoding the viral coat. The conditioned media containing lentivirus was collected the next day and stored at −20°C. The process was repeated for 2 additional days. Finally, conditioned media were centrifuged at 1,000g for 3 min and the supernatant was passed through a 0.45 μm filter to make lentivirus infection media.

### shRNA lentivirus infection

SH-SY5Y cells were plated in 10 cm dish–one for scramble and one for each target shRNA. The next day, culture medium was discarded in each plate and replaced with 4 mL of fresh medium and 4 mL lentivirus for infection. Culture medium in each plate was discarded the next day and replaced with 10 ml fresh medium supplied with puromycin at 1 μg/ml. The process was continued for the next 4–9 days.

### Animal protocols and models

The University of Kansas Medical Center Animal Care and Use Committee approved all experiments in this study. Two-month-old male C57BL/6J and 5XFAD mice were purchased from The Jackson Laboratory (Bar Harbor, ME). All mice were housed using a standard 12-h light/dark cycle with access to chow and water *ad libitum*. Mice were treated with a 50 mg/kg TMG intraparietal injection every other morning for 1 month or 6 months (Macauley and Vocadlo, 2010).

### Lysis and immunoblotting

Cells were lysed on ice in Np-40 lysis buffer (containing 20 mM Tris, pH 7.4, 150 mM NaCl, 40 mM GlcNAc, 2 mM EDTA, 1 mM DTT, 1% Non-idet P-40 with phosphatase inhibitors 1 mM β-glycerophosphate, 1 mM Sodium Fluoride (NaF), and protease inhibitors 2 mM phenylmethylsulfonyl fluoride (PMSF) and 1 × inhibitor mixture composed of 1 μg/ml leupeptin, 1 μg/ml antipain, 10 μg/ml benzamidine, and 0.1% aprotinin added immediately before lysis). Animal tissues were lysed with RIPA buffer (containing 10 mM Tris, pH 7.6, 150 mM NaCl, 40 mM GlcNAc, 2 mM EDTA, 1 mM DTT, 1% Non-idet P-40, 0.1% SDS, 0.5% deoxycholic acid with phosphatase inhibitors 1 mM β-glycerophosphate, 1 mM Sodium Fluoride (NaF), and protease inhibitors 2 mM phenylmethylsulfonyl fluoride (PMSF) and 1 × inhibitor mixture composed of 1 μg/ml leupeptin, 1 μg/ml antipain, 10 μg/ml benzamidine, and 0.1% aprotinin added immediately before lysis). Lysates were incubated on ice for 20 min and vortexed every 5 min. Protein concentration of the lysate was determined using Bradford assay (Bio-Rad Catalog), or BCA assay (ThermoFisher). Lysates were then denatured by the addition of 4 × protein solubility mixture (100 mM Tris, pH 6.8, 10 mM EDTA, 8% SDS, 50% sucrose, 5% β-mercaptoethanol, 0.08% Pyronin Y) and boiling for 2 min. Equal protein amounts of lysates were loaded onto 4–15% Criterion precast TGX gels (Bio-Rad). Electrophoresis occurred at 130 V for about 50 min, and then the gel proteins were transferred to polyvinylidene difluoride (PVDF) membranes at 0.4 Amps. Membranes were blocked with 3% BSA, 0.01% sodium azide in TBST (25 mM Tris, pH 7.6, 150 mM NaCl, 0.05% Tween 20) for at least 20 min. Blots were then probed overnight at 4°C with primary antibody to the protein of interest at 1:1,000 dilution. The next day, blots were washed three times in TBST for 10 min each. HRP-conjugated secondary antibody (Bio-Rad) at 1:10,000 dilution was added for 1 h at room temperature, followed by washing three times for 10 min each. Blots were then developed using the chemiluminescence HRP antibody detection method (ThermoFisher Catalog # 34095). Blots were striped with 200 mM glycine, pH 2.5, for 1 h at room temperature, blocked, and re-probed. Where shown, ImageJ 3.2 (National Institutes of Health) or image Lab (Biorad) was used to quantify the density of protein bands compared with an internal standard protein band such as actin or α-Tubulin. At least three independent experiments were repeated for all immunoblotting (Tan et al., 2017; Machacek et al., 2018).

### Antibodies

All primary and secondary antibodies used for immunoblotting were used at a concentration of 1:1000 and 1:10,000 dilution accordingly. Antibodies used in these studies were as follows: *O*-GlcNAc (RL2, ThermoFisher #MA1-072), actin (Sigma Catalog # A5316), α-Tubulin (sigma Catalog # T5168), OGT (AL-34) and OGA (345) antibodies were a generous gift from the laboratory of Dr. Gerald Hart (Department of Biological Chemistry, University of Georgia), Phospho-ERK (CST Catalog #9101), Total-ERK (Santa Cruz, Catalog # sc-93), phospho-MEK (ThermoFisher Catalog # 44-454G), Total MEK (ThermoFisher Catalog # 13-3500), APP (CST Catalog # 2452), and DUSP4 (CST Catalog # 5149).

### Statistical analysis

Statistical significance of all results was assessed using *t*-test (paired for cells and un-paired for mice) with *p* < 0.05 considered to be statistically significant. All data were generated using at least three independent experiments (Tan et al., 2017).

## Results

### Long-term OGA inhibition increases ERK phosphorylation

**FIGURE 2:**
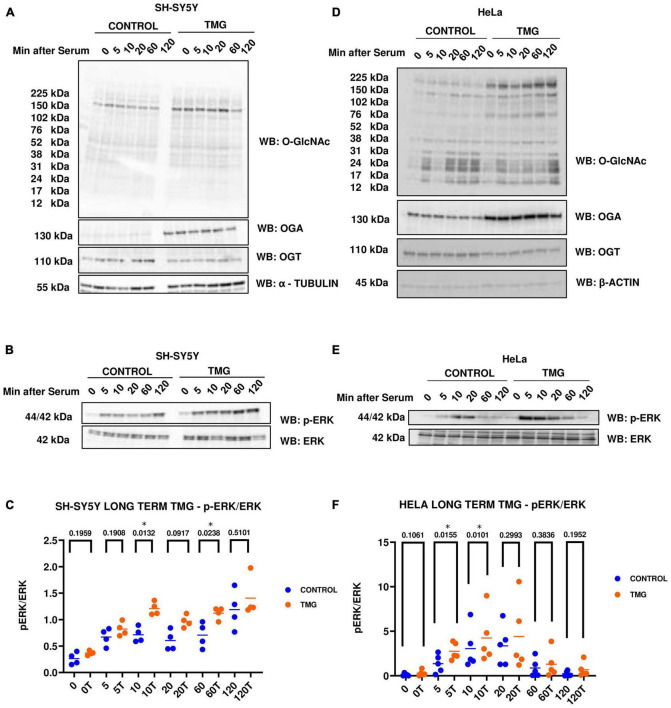
Western blot analysis of samples harvested after serum reactivation time course in panels **(A,B)** SH-SY5Y neuroblastoma cells and **(D,E)** HeLa cervical cancer cells subjected to long term OGA inhibition with TMG. **(C,F)** Densitometry plot of ERK 1/2 phosphorylation normalized to total ERK 1 in SH-SY5Y cells (*n* = 4) and HeLa (*n* = 5), where n = total number of experimental trials. Dots represent the number of experimental trials (n). Statistical significance was measured using paired-*t*-test analysis and *p*-value is indicated on the plots. *Is added for *p*-values that are significant (*p* < 0.05).

Previously, Ingenuity pathway analysis of RNA Sequencing data from long-term OGA inhibition by Thiamet-G (TMG) in SH-SY5Y neuroblastoma cells predicted that ERK signaling is significantly up-regulated (Tan et al., 2017). To validate the findings, we used two different cell lines, namely, SH-SY5Y neuroblastoma and HeLa cervical cancer cells, subjected to long-term TMG treatment (minimum of 14 days). We used two different cell lines to determine if the O-GlcNAc induced increase in ERK phosphorylation was cell-type specific or a phenotype that occurs across different cell lines. These cells were serum starved overnight and re-stimulated the next day to induce a robust activation of ERK. Cells were harvested at 0, 5, 10, 20, 60, and 120 min upon serum re-activation, and western blot analysis was done to measure ERK activation by phosphorylation. We found that O-GlcNAc and OGA increases with long-term TMG treatment in both SH-SY5Y neuroblastoma cells (Figure 2A) and HeLa cervical cancer cells (Figure 2D) while OGT decreases as expected. The amplitude of ERK phosphorylation (p-ERK) significantly increases with long-term TMG treatment in both SH-SY5Y and HeLa cells compared to control (Figures 2B, C, E, F). These data indicate that long-term OGA inhibition amplifies ERK phosphorylation in both SH-SY5Y neuroblastoma and HeLa cervical cancer cells.

### OGT KD increases ERK phosphorylation

**FIGURE 3:**
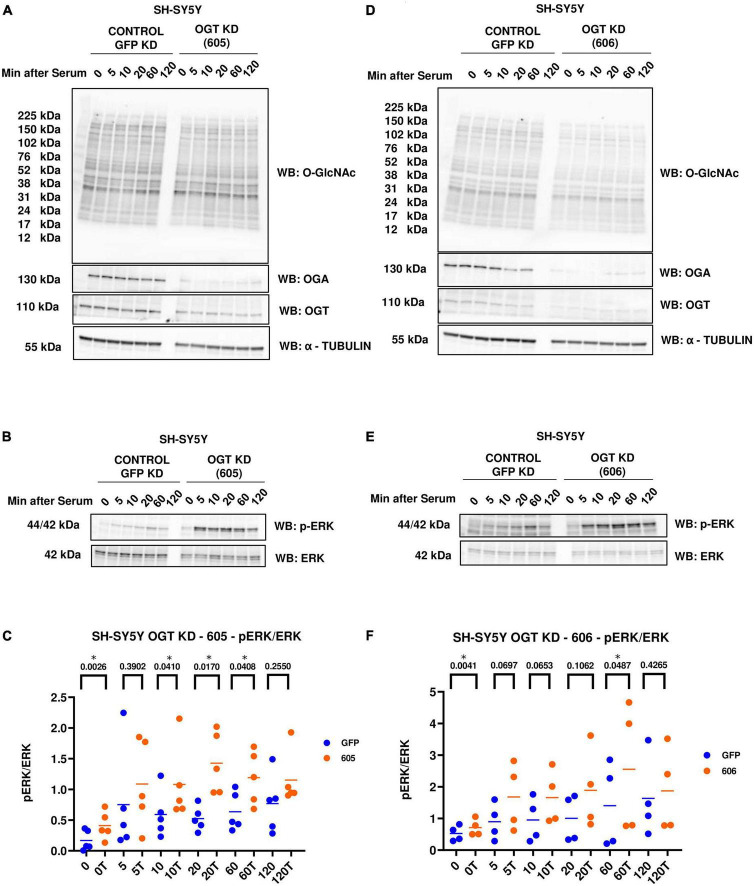
Western blot analysis of samples harvested after serum reactivation time course of SH-SY5Y OGT KD cells **(A,B)** 605 and **(D,E)** 606. **(C,F)** Densitometry plot of ERK 1/2 phosphorylation normalized to total ERK 1 in SH-SY5Y cells with **(C)** OGT KD 605 (*n* = 5) and **(F)** OGT KD 606 (*n* = 4), where n = total number of experimental trials. Dots represent the number of experimental trials (n). Statistical significance was measured using paired-*t*-test analysis and *p*-value is indicated on the plots. *Is added for *p*-values that are significant (*p* < 0.05).

Next, we evaluated the effect of genetic manipulation of O-GlcNAc on ERK activation. We did a similar serum-reactivation time-course with SH-SY5Y OGT KD 605 and 606 cells (where 605 and 606 are two different OGT KD short-hairpin shRNAs). OGT KD caused O-GlcNAc to decrease as expected. OGA also decreased with OGT knockdown (Figures 3A, D). Interestingly, we saw that OGT KD amplifies p-ERK signal like TMG treatments (Figures 3B, C, E, F).

### OGA KD increases ERK phosphorylation

**FIGURE 4:**
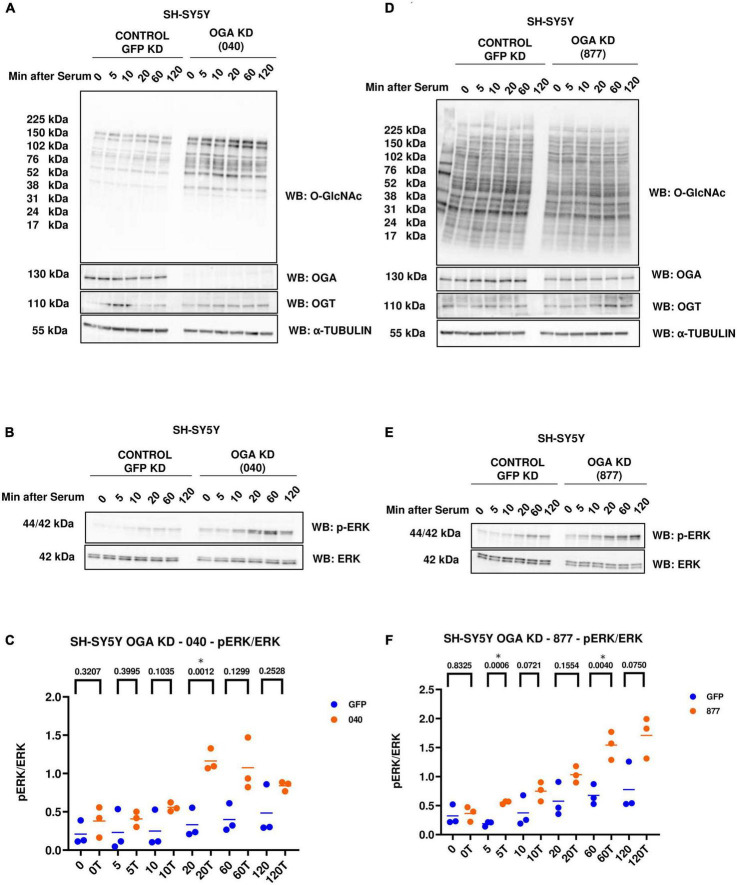
Western blot analysis of samples harvested after serum reactivation time course of SH-SY5Y OGA KD cells **(A,B)** 040 and **(D,E)** 877. **(C,F)** Densitometry plot of ERK 1/2 phosphorylation normalized to total ERK 1 in SH-SY5Y cells with **(C)** OGA KD 040 (*n* = 3) and **(F)** OGA KD 877 (*n* = 3), where n = total number of experimental trials. The dots represent the number of experimental trials (n). Statistical significance was measured using paired-*t*-test analysis and *p*-value is indicated on the plots. *Is added for *p*-values that are significant (*p* < 0.05).

In addition to OGT KD cells, we also did a serum-reactivation time-course with SY5Y OGA KD 040 and 877 cells (where 040 and 877 are two different OGA KD short-hairpin shRNAs). O-GlcNAc increases and OGA decreases as expected with OGA KD. There is no change in OGT levels (Figures 4A, D). OGA KD amplified p-ERK activation like TMG treatment and OGT KD (Figures 4B, C, E, F).

### O-GlcNAc increases ERK phosphorylation via changes to MEK and DUSP4

**FIGURE 5:**
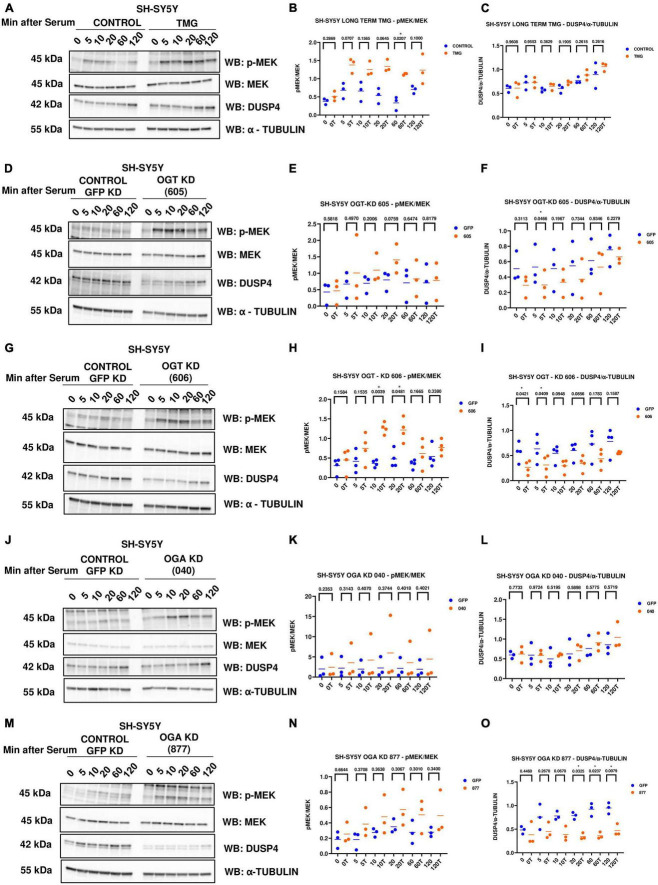
Western blot analysis of samples harvested after serum reactivation time course of SH-SY5Y cells treated with **(A)** TMG as in Figure 1, or subjected to panel **(D)** OGT KD 605, **(G)** OGT KD 606, **(J)** OGA KD 040, and **(M)** OGA KD 877. The blots were probed for p-MEK 1/2, MEK 1/2, Dusp4 and α-Tubulin. **(B,E,H,K,N)** Densitometry plot of MEK 1/2 phosphorylation normalized to total MEK 1/2 in SH-SY5Y cells with **(B)** TMG (*n* = 3), **(E)** OGT KD 605 (*n* = 4), **(H)** OGT KD 606 (*n* = 4), **(K)** OGA KD 040 (*n* = 3), and **(N)** OGA KD 877 (*n* = 3). Densitometry plot of DUSP4 normalized to α-tubulin in SH-SY5Y cells with **(C)** TMG (*n* = 3) **(F)** OGT KD 605 (*n* = 4) **(I)** OGT KD 606 (*n* = 4) **(L)** OGA KD 040 (*n* = 3), and **(O)** OGA KD 877 (*n* = 3), where n = total number of experimental trials. The dots represent the number of experimental trials (n). Statistical significance was measured using paired-*t*-test analysis and *p*-value is indicated on the plots. *Indicates *p*-values that are significant (*p* < 0.05).

Phosphorylated Mitogen-activated protein kinase kinase (p-MEK) phosphorylates and activates ERK. Dual specificity phosphatases (DUSPs) deactivate ERK by removing the phosphate group from ERK. Next, we wanted to see if O-GlcNAc regulates these upstream kinases and downstream phosphatases that regulate ERK phosphorylation. We did serum reactivation time-course with long-term TMG-treated, OGT KD, and OGA KD SH-SY5Y cells and measured p-MEK and DUSP4 (previously identified in the RNA sequencing data) levels. We found that p-MEK increases and DUSP4 does not change with long-term TMG treatment (Figures 5A–C). p-MEK increases and DUSP4 decreases upon OGT KD with both shRNA 605 (Figures 5D–F) and 606 (Figures 5G–I). However, the increase in p-MEK was not statistically significant with OGT KD 605. p-MEK increases but is not statistically significant upon OGA KD with shRNA 040 (Figures 5J, K) and 877 (Figures 5M, N). However, DUSP4 does not change upon OGA KD with shRNA 040 (Figures 5J, L) while DUSP4 significantly decreases upon OGA KD with shRNA 877 (Figures 5M, O).

### Long-term OGA inhibition increases ERK phosphorylation and APP in C57BL6/J mice brains

**FIGURE 6:**
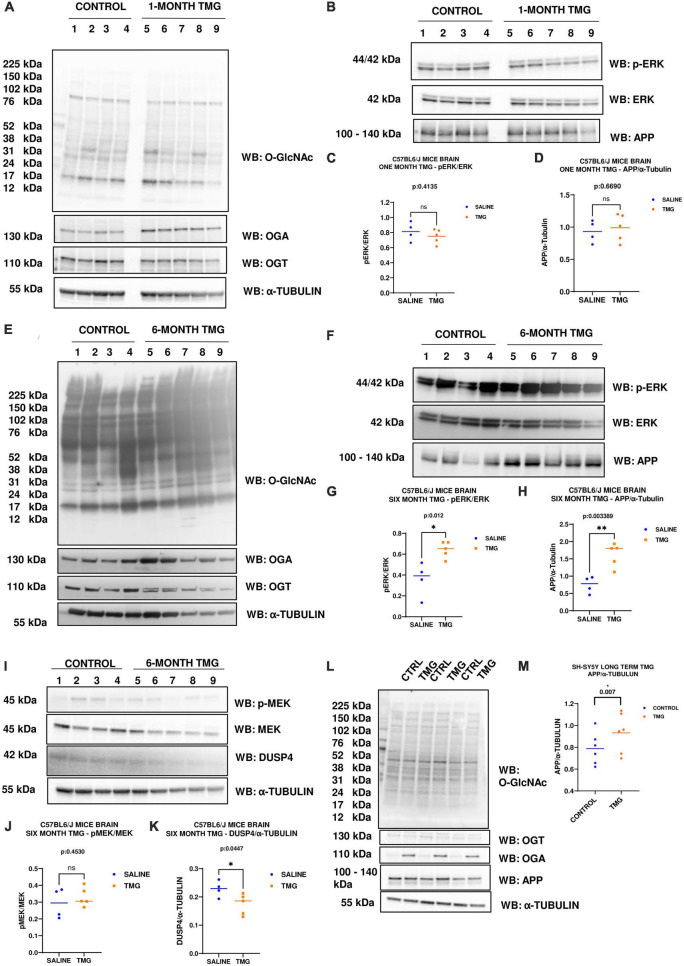
**(A–D)** Western blot analysis of total brain lysate of WT-C57BL/6J male mice subjected to intra-peritoneal TMG injection for 1 month. The blots were probed for panel **(A)** O-GlcNAc, OGT, OGA, α-Tubulin **(B)** p-ERK 1/2, Total ERK 1, and APP. **(C)** Densitometry plot of ERK 1/2 phosphorylation normalized to total ERK 1 and **(D)** APP normalized to α-Tubulin. The dots represent the number of mice (*n* = 4 for control and *n* = 5 for TMG injected mice). Statistical significance was measured using un-paired-*t*-test analysis and *p*-value is indicated on the plots. **(E–K)** Western blot analysis of total brain lysate of WT-C57BL/6J male mice subjected to intra-peritoneal TMG injection for 6 months. The blots were probed for panel **(E)** O-GlcNAc, OGT, OGA, α-Tubulin **(F)** p-ERK 1/2, Total ERK 1, and APP **(I)** p-MEK 1/2, MEK 1/2, Dusp4 and α-Tubulin. Densitometry plot of panel **(G)** ERK 1/2 phosphorylation normalized to total ERK 1 **(H)** APP normalized to α-Tubulin. **(J)** MEK 1/2 phosphorylation normalized to total MEK 1/2 and **(K)** DUSP4 normalized to α-tubulin. The dots represent the number of mice (*n* = 4 for control and *n* = 5 for TMG injected mice). Statistical significance was measured using un-paired-*t*-test analysis and *p*-value is indicated on the plots. **(L,M)** Western blot analysis of SH-SY5Y neuroblastoma cells subjected to long term OGA inhibition with TMG treatment. The blots were probed for panel **(L)** O-GlcNAc, OGT, OGA, APP, α-Tubulin. **(M)** Densitometry plot of APP normalized to α-Tubulin. The dots represent the number of the number of experimental trials (n). Statistical significance was measured using paired-*t*-test analysis and *p*-value is indicated on the plots. **Indicates *p*-values that are significant and less than 0.01 (*p* < 0.01).

Since increased ERK phosphorylation is associated with AD pathology, we further evaluated the effect of O-GlcNAc mediated increased ERK phosphorylation *in vivo* using the brains of WT-C57BL/6J male mice intraperitoneally injected for 1 month with TMG. Brains were harvested, homogenized and western-blot analysis was done. There was no change in the amplitude of ERK phosphorylation in the brains of WT-C57BL/6J mice after 1-month TMG injection (Figures 6A–D). Next, we extended the injections for a period of 6 months in WT-C57BL/6J mice to simulate a long-term inhibition of OGA. We found that phosphorylated ERK and APP increased with 6-month TMG injection compared to control (Figures 6E–H). With 6-month TMG injection compared to control mice, the increase in p-MEK was not significant, however, DUSP4 decreased significantly (Figures 6I–K). Next, to investigate if the increase in APP protein levels occurs via p-ERK, we harvested SH-SY5Y neuroblastoma cells without serum reactivation. APP significantly increased in SH-SY5Y cells with TMG treatment (Figures 6L, M).

### Long-term OGA inhibition increases ERK phosphorylation in 5XFAD AD mice brains

**FIGURE 7:**
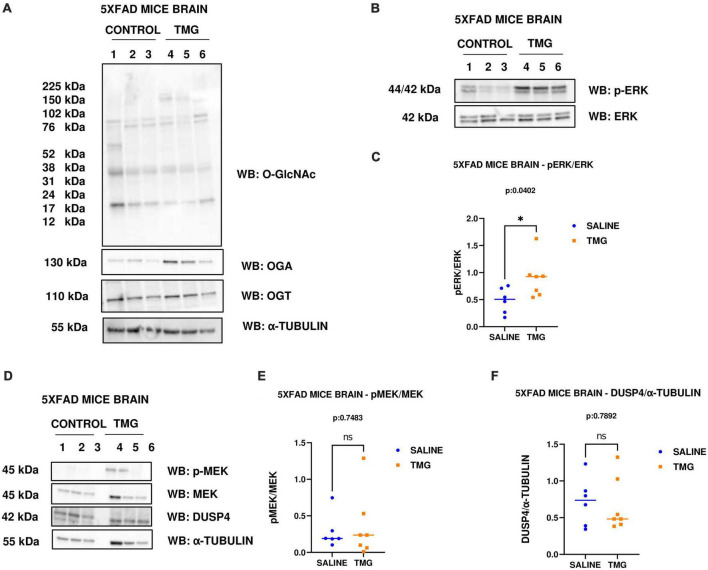
Western blot analysis of total brain lysate of 5XFAD mice subjected to intra-peritoneal TMG injection for 1 month. The blots were probed for panel **(A)** O-GlcNAc, OGA, OGT, α-Tubulin **(B)** p-ERK 1/2, Total ERK 1, and APP **(D)** p-MEK 1/2, MEK 1/2, Dusp4 and α-Tubulin. Densitometry plot of panel **(C)** ERK 1/2 phosphorylation normalized to total ERK 1 **(E)** MEK 1/2 phosphorylation normalized to total MEK 1/2 and **(F)** DUSP4 normalized to α-tubulin in 5XFAD mice brains. The dots represent the number of mice (*n* = 6 for control and *n* = 7 for TMG injected mice). Statistical significance was measured using un-paired-*t*-test analysis and *p*-value is indicated on the plots. *Indicates *p*-values that are significant (*p* < 0.05).

Since OGA inhibition is being considered for long-term AD treatment, we replicated OGA inhibition for 1 month in the 5XFAD Alzheimer’s disease mice which express high APP protein levels. With 1-month intra-peritoneal TMG injection, OGA increased and there was no change in O-GlcNAc and OGT levels in the 5XFAD mice brains. Interestingly, phosphorylated ERK increased with 1-month TMG injection compared to control (Figures 7A–C). However, the increase in p-MEK and decrease in DUSP-4 was not statistically significant (Figures 7D–F).

## Discussion

**FIGURE 8:**
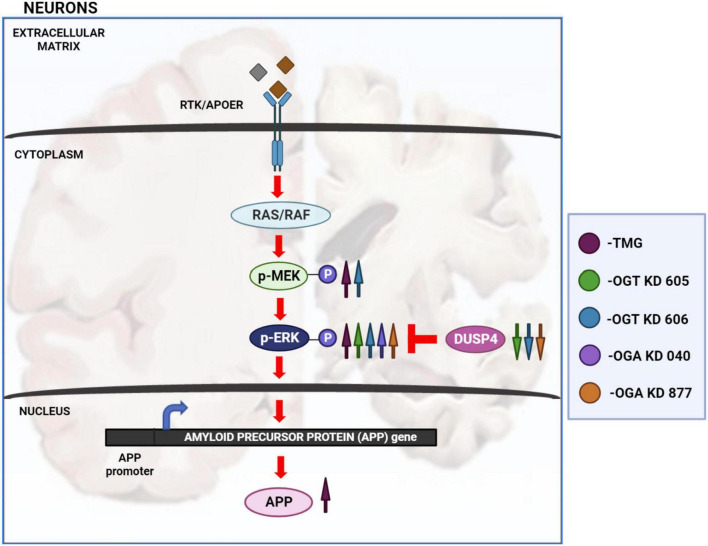
Changes in O-GlcNAcylation amplify ERK 1/2 signaling. Arrows in magenta indicate long term OGA inhibition via TMG treatment for at least 2 weeks. Green arrows indicate OGT KD via shRNA 605. Blue arrows indicate OGT KD via shRNA 606. Purple arrows indicate OGA KD via shRNA 040. Orange arrows indicate OGA KD via shRNA 877. OGA inhibition via long term TMG treatment, OGT KD and OGA KD, all increase the amplitude of ERK 1/2 phosphorylation. TMG treatment increases ERK 1/2 phosphorylation by increasing the amplitude of MEK 1/2 phosphorylation. The levels of DUSP4 does not change with TMG treatment. OGT KD 606 also increases ERK 1/2 phosphorylation by increasing the amplitude of MEK 1/2 phosphorylation. The levels of DUSP4 decrease with OGT KD 606. OGT KD with shRNA 605 follows a similar trend as OGT KD with shRNA 606 where the levels of DUSP4 decreases. But the increase in MEK 1/2 phosphorylation by OGT KD 605 is not significant. OGA KD with both shRNA 040 and 877 increase ERK 1/2 phosphorylation but increase in the level of MEK 1/2 phosphorylation was not significant. DUSP4 decreases significantly with OGA KD 877 and DUSP4 levels do not change with OGA KD 040. Long term OGA inhibition also increased APP protein levels, correlating with increased ERK phosphorylation.

Disruptions to O-GlcNAc homeostasis and ERK hyper-activation are both associated with AD pathogenesis (Griffith and Schmitz, 1995; Förster et al., 2014; Huang et al., 2017). Our study reveals pharmacological and genetic disruption of O-GlcNAc homeostasis amplifies ERK phosphorylation, indicating a significant role for O-GlcNAc in regulating ERK activation. We found that OGA inhibition via long-term TMG treatment, OGT KD, and OGA KD all increase the amplitude of ERK1/2 phosphorylation. Additionally, 6-month TMG injection in C57BL/6J mice showed an increase in amplitude of ERK phosphorylation and APP protein levels. Long term OGA inhibition also increased APP protein levels in SH-SY5Y neuroblastoma cells. Furthermore, 1-month TMG injection was sufficient to increase the amplitude of ERK phosphorylation in 5XFAD AD mice brains. The O-GlcNAc mediated increase in ERK1/2 activation was found to occur via the increased phosphorylation of upstream kinases such as MEK and reduced expression of downstream phosphatases like DUSP4 (Figure 8). These results indicate that O-GlcNAc mediated changes to MEK phosphorylation or DUSP4 expression lead to amplification of ERK phosphorylation, which correlates with increased APP protein levels.

At least two other studies have reported that O-GlcNAc regulates ERK signaling. First, MEK and ERK phosphorylation increased after OGT KD in Mouse Embryonic Stem Cells (MESCs). OGT KD reduced O-GlcNAc on Protein kinase C ζ (PKC ζ) leading to increased phosphorylation and activation of PKC ζ. Active PKC ζ phosphorylates MEK, which phosphorylates and activates ERK1/2 to inhibit the maintenance of the undifferentiated state of MESCs. MEK phosphorylation was increased in MESCs without stimulus suggesting that OGT KD stimulates baseline PKC ζ signaling (Miura et al., 2018). In contrast to the MESCs study and our study, OGT KD in MDA-MB-231, MDA-MB-468, and MDA-MB-157 breast cancer cells increased SIRT1 levels and activity causing a reduction of MEK/ERK signaling, increased proteasomal degradation, and decreased activity of the oncogenic transcription factor FOXM1, and lowered invasion potential (Ferrer et al., 2017). However, our data implies that the regulation of ERK-activation by O-GlcNAc could be different in AD and embryonic stem cells compared to metastatic breast cancer. This could be because metastatic breast cancer cells have altered genomes due to mutations, insertion/deletions, and gene duplications that could affect O-GlcNAc mediated ERK pathway activation (Yates et al., 2017; Hu et al., 2022). Recently, overexpression of constitutively active form of ERK homolog (rl^Sem^) in *D. melanogaster* decreased neuronal responses to sucrose and this effect was blocked by OGT inhibition with OSMI suggesting that a nutrigenomic pathway composed of ERK signaling upstream of OGT regulates sensory responses to diet (Sung et al., 2023). Thus, they show that OGT activity is regulated by ERK signaling in contrast to our data showing disruptions to O-GlcNAc regulates ERK activation.

Of note, MEK activation is implicated in AD pathogenesis, where MEK activation is significantly up-regulated leading to increased ERK activation in AD, and MEK inhibitors decrease Aβ plaque formation in SY5Y cells and 5XFAD AD mice models (Mills et al., 1997; Zhu et al., 2003; Araki et al., 2010; Chun et al., 2022). Our data supports these results suggesting that altered O-GlcNAc homeostasis seen in AD can activate ERK. However, how disrupted O-GlcNAc changes amplify MEK to activate ERK in AD is unknown. O-GlcNAc changes could occur either via post-translational modifications on MEK and ERK or other kinases. In undifferentiated ESCs, MEK is not O-GlcNAcylated (Miura et al., 2018) but MEK was recently shown to be O-GlcNAcylated in neonatal rat ventricular myocyte (NRVM) via metabolic labeling using the sugar analogs Ac4GalNAz or Ac4GalNAlk (Papanicolaou et al., 2023). Interestingly, ERK is also O-GlcNAcylated (Dehennaut et al., 2008; Papanicolaou et al., 2023). However, MEK O-GlcNAcylation and ERK O-GlcNAcylation in AD is unclear; the O-GlcNAc sites on MEK and ERK have not been mapped in AD tissue. Thus, ERK and MEK could be a target of O-GlcNAcylation in AD as well; hence, further experiments are needed to clarify the role of O-GlcNAc on ERK and MEK.

The ERK1/2 phosphatase DUSP-4 is important to maintain proper neuronal functions and is down-regulated in AD pathogenesis. DUSP-4 deletion in mice impairs cognition and memory (Rahman et al., 2016) and overexpressing DUSP4 reduced amyloid plaque burden in the hippocampi of 5XFAD AD mice brains (Beckmann et al., 2020; Pan et al., 2022). Our data supports these findings and suggests that disrupted O-GlcNAcylation upregulates the amplitude of ERK activation by down-regulating DUSP-4, contributing to AD pathogenesis. Specifically, DUSP4 protein levels decreased with long term (6-month) OGA inhibition in C57BL6/J mice brains, along with OGT KD and OGA KD in SH-SY5Y cells suggesting that the O-GlcNAc mediated decrease in DUSP-4 could either be due to the regulation of DUSP4 gene expression or protein stability (Zhang et al., 2014). Thus, the combination of increased MEK1/2 phosphorylation and lower DUSP4 expression caused by disrupted O-GlcNAcylation amplifies ERK1/2 signaling, potentially contributing to AD pathogenesis. These data are clinically important since several OGA inhibitors are in clinical trials for AD treatments (Selnick et al., 2019; Shcherbinin et al., 2020; Permanne et al., 2022).

AD brains have increased tau phosphorylation that could be reversed by OGA inhibition, where OGA inhibition increases tau O-GlcNAcylation and prevents phosphorylation at the pathological tau phosphorylation sites (Yuzwa et al., 2008, 2012; Zhu et al., 2014). OGA inhibitors such as MK-8719 from Merck/Alectos, ASN-120,290 from Asceneuron S.A., and LY-3,372,689 from Eli Lilly are currently in Phase I or II clinical trials to increase O-GlcNAcylation of tau and prevent tau phosphorylation for the treatment of AD (Selnick et al., 2019; Shcherbinin et al., 2020; Permanne et al., 2022). Although we did not see any changes to tau phosphorylation, 1-month TMG injection was sufficient to increase ERK phosphorylation in 5XFAD Alzheimer’s disease mice brains compared to 6-month injection in C57BL6/J mice. Potentially, the AD phenotype of 5XFAD mice accelerates the O-GlcNAc mediated increase in ERK activation. Furthermore, 6-month TMG injection increased APP levels in addition to ERK phosphorylation in C57BL6/J mice brains. Long term OGA inhibition also increased APP protein levels in SH-SY5Y neuroblastoma cells. These data indicate that increased ERK phosphorylation correlates with increased APP protein levels, potentially affecting Aβ plaque formation and accelerating AD. Therefore, we argue that administering OGA inhibitors to patients for AD treatment needs further evaluation, especially an assessment for the risk of activating ERK signaling.

## Data availability statement

The raw data supporting the conclusions of this article will be made available by the authors, without undue reservation.

## Ethics statement

The animal study was reviewed and approved by the IACUC Committee University of Kansas Medical Center.

## Author contributions

SE conducted the experiments including cell-culture, western-blot, 6-month mice injections, and drafted all sections of the manuscript and figures. GC, VM, JS, and MJ generated the experimental replicates. IA and AQ helped with the 6-month mice injections. IA also helped to generate SH-SY5Y OGA KD cells. HF maintained the mice colonies. AD and MC did the 1-month mice injections. RS provided the guidance on AD studies. CS mentored the research and edited the all sections of the manuscript and figures. All authors contributed to the article and approved the submitted version.
